# A pattern of unspecific somatic symptoms as long-term premonitory signs of type 2 diabetes: findings from the population-based MONICA/KORA cohort study, 1984-2009

**DOI:** 10.1186/1472-6823-14-87

**Published:** 2014-11-22

**Authors:** Jens Baumert, Christa Meisinger, Karoline Lukaschek, Rebecca Thwing Emeny, Ina-Maria Rückert, Johannes Kruse, Karl-Heinz Ladwig

**Affiliations:** Institute of Epidemiology II, Helmholtz Zentrum München, German Research Center for Environmental Health, Ingolstädter Landstr. 1, Neuherberg, 85764 Germany; Central Hospital of Augsburg, MONICA/KORA Myocardial Infarction Registry, Augsburg, Germany; Department of Psychosomatic Medicine and Psychotherapy, University of Giessen, Giessen, Germany; Department of Psychosomatic Medicine and Psychotherapy, University of Marburg, Marburg, Germany; Department of Psychosomatic Medicine and Psychotherapy, Klinikum rechts der Isar, Technische Universität München, Munich, Germany

**Keywords:** Cohort study, General population, Type 2 diabetes mellitus, Unspecific premonitory symptom pattern

## Abstract

**Background:**

Unspecific symptoms often proceed a serious chronic disease condition long before the onset of the disease. The role of an unspecific premonitory symptom (UPMS) pattern as premonitory signs of subsequent type 2 diabetes mellitus (T2DM) diagnosis independent of established cardio-metabolic risk factors is unclear and therefore was examined in the present study.

**Methods:**

The study population consisted of 10,566 participants aged 25–74 years at baseline drawn from the population-based MONICA/KORA Cohort Study conducted in 1984-2009 in the Augsburg region (Germany). Unspecific premonitory symptoms were assessed following the Somatic Symptom Scale-8 (SSS-8). The impact of the score on T2DM risk within a mean follow-up time of 16 years was estimated by Cox regression.

**Results:**

Within follow-up, 974 newly diagnosed T2DM cases were observed. The risk for T2DM increased by a hazard ratio (HR) of 1.03 (95% CI 1.01-1.04, p value < 0.001) for a one unit increase of the UPMS score in a Cox model adjusted for age, sex and survey. Additional adjustment for cardio-metabolic risk factors attenuated this effect (HR = 1.02) but significance remained (p value = 0.01).

**Conclusions:**

Suffering from an elevated burden of unspecific somatic symptoms is associated with T2DM long before the onset and independent of established cardio-metabolic risk factors. Further research is needed to obtain insight in potential underlying pathophysiological mechanisms.

## Background

An unspecific premonitory symptom (UPMS) pattern is often seen in clinical practice as a prodromal sign of a serious chronic disease condition long before the onset of the disease. In case of an acute coronary syndrome, it is well established that symptoms of unusual fatigue, dizziness and vital exhaustion several months preceding the event are very common [1, 2]. However, an UPMS pattern is also prevalent among subjects with no obvious medical disorder [3]. Often, the medical significance of the symptoms that are reported is unclear and an assignment to a specific underlying disease is not always possible [4, 5]. In primary care, about half of the symptoms reported may be functional [6].

One major obstacle in the treatment of type 2 diabetes mellitus (T2DM) is the well acknowledged fact that the disease often remains undiagnosed for many years. A progressive decrease in ß-cell insulin secretion begins as early as 12 years before diagnosis [7]. The onset of an increased risk to develop micro- and macrovascular complications also predates the point of clinical recognition by several years [8, 9] such that at diagnosis approximately 50% of patients have evidence of diabetes-related complications [9, 10]. However, the disease condition often remains being viewed as asymptomatic in its early stages [11].

Premonitory signs before the onset of T2DM are mainly related to hyperglycaemic states and acute metabolic disturbances. Here, polyuria, polydipsia, weight loss, sometimes along with polyphagia, and blurred vision are acknowledged as key symptoms. Subjects suffering from these symptoms are viewed being at increased risk to experience the onset of T2DM [12]. The American Diabetes Association (ADA) and the International Diabetes Federation (IDF) developed lists of symptoms [13, 14] which are recommended to call for immediate medical attention if individuals experience one or more of these symptoms. Although unusual fatigue and irritability as unspecific mood changes are also taken into consideration, to the best of our knowledge, no investigation to date has studied whether a more unspecific symptom picture beyond the known premonitory signs of an acute hyperglycaemia is indicative for the development of T2DM several years before the point of clinical recognition.

Thus, the aim of the present study was to assess the role of an unspecified premonitory pattern on newly diagnosed T2DM in a large prospective study including men and women from the general population, independent of established cardio-metabolic lifestyle and mental health related risk factors. Furthermore, we analysed whether this association might be modified by these classic risk factors.

## Methods

### Study design

The data of the present study were drawn from the population-based MONICA/KORA Augsburg cohort study based on three independent surveys (S1, S2, S3) carried out from 1984 to 1995 in the Augsburg region, southern Germany, including 13,426 participants aged 25-74 years [15]. The MONICA (MONItoring of trends and determinants in CArdiovascular disease) Augsburg project was part of the multinational WHO MONICA project [16]. The incidence of type 2 diabetes was determined within the framework of the KORA (Cooperative Health Research in the Region of Augsburg) platform by follow-up examinations until 2009 [17]. The study followed the declaration of Helsinki and was approved by the local authorities: The MONICA surveys S1, S2 and S3 with the baseline examination were approved by the data protection commission following the rules at the time of the examinations (1984/85, 1989/90 and 1994/95); the follow-up examinations within the KORA framework afterwards were approved by the ethics committee of the Bavarian Medical Association ("Bayerische Landesärztekammer"). Written informed consent was obtained from each study participant.

### Study population

Among the whole MONICA/KORA Augsburg cohort study (n = 13,426), psychosocial data using a self-administered questionnaire were assessed in 12,886 participants. The psychosocial data set extended the MONICA core design and followed recommendations given by the MONICA steering committee [18]. Among them, participants with a self-reported history of diabetes at baseline examination (n = 535), missing information on newly diagnosed type 2 diabetes (n = 626), unspecific premonitory symptom patterns (n = 840), or any other variable described below (n = 319) were excluded from the present analyses leading to a study population of 10,566 participants (5,448 men, 5,118 women) with a mean age of 46.7 years (standard deviation (SD) 13.2) ranging from 25 to 74 years at baseline. The mean follow-up time for the present study population was 15.6 years with a standard deviation of 6.2 and a range from 0.1 to 25.2 years in 164,963 person-years.

### Definition of an unspecified premonitory symptom pattern

The unspecified premonitory symptom pattern was assessed at the baseline examination by creating a score based on similar items of the recently developed 8-item Somatic Symptom Scale-8 (SSS-8) which shows high reliability and validity [19, 20]. The following eight items from the von Zerssen symptom checklist [21] were used to build our modified SSS-8: stomach or bowel pain, back pain, pain in the joints, headaches or pressure in the head, temporary shortness of breath, dizziness, feeling tired and insomnia. Each item was measured on a four-point scale ranging from 0 (not present) to 3 (strong) leading to an UPMS score ranging from 0 to 24 (the items of SSS-8 are measured on a five-point scale) which was approximately normal distributed. Cronbach’s α was estimated as 0.75 in the present study indicating a good reliability. For descriptive purposes, the UPMS score was classified into three categories using tertiles as cut-off points.

### Definition of newly diagnosed type 2 diabetes

A written follow-up questionnaire was sent to all participants of the three baseline surveys in 1997/1998, in 2002/2003 and in 2008/2009. Furthermore, all subjects who participated in the first survey were invited to participate in a follow-up examination conducted in 1987/1988. Self-reported newly diagnosed cases of diabetes mellitus and the date of diagnosis were validated by hospital records or by contacting the proband’s treating physician. Furthermore, the hospital records of those deceased during the follow-up period without a diagnosis of type 2 diabetes mellitus at baseline were also examined and/or their last treating physicians were contacted. The records were searched for or the doctors were asked for a history concerning diabetes and if a person had suffered from diabetes, the type of diabetes and the date of diagnosis were ascertained. If a participant was not found in any of the medical records and if no information from the last treating physician could be obtained, the participant was excluded from analysis [22].

### Definition of cardio-metabolic risk factors

Standardized face-to-face interviews were conducted at baseline examination by trained medical staff (mainly nurses) to assess information concerning sociodemographic, lifestyle and clinical characteristics. Additionally, participants underwent an extensive standardised medical examination including collection of a non-fasting venous blood sample. All assessment procedures followed the standardized protocol of the WHO MONICA project [16] and have been described in detail elsewhere [23, 24].

Low educational level was defined as having less than 12 years of schooling. Parental history of diabetes was classified as ‘yes’ if the participant reported that at least one of the parents had diabetes, ‘no’ if no diabetes for both parents was reported and ‘unknown’ in all other cases.

Current smoking was defined as currently smoking at least one cigarette per day or smoking occasionally. Alcohol consumption was classified into three categories: none (0 g/day), moderate (0.1-39.9 g/day for men and 0.1-19.9 g/day for women) and high (≥40.0 g/day for men and ≥20.0 g/day for women). To assess physical activity, participants were classified as ‘active’ during leisure time if they regularly participated in sports for at least 1 hour per week; otherwise they were considered ‘inactive’.

Actual hypertension was defined as blood pressure ≥140/90 mmHg and/or current use of hypertensive medication, given that the subjects were aware of being hypertensive. Total cholesterol (TC) and high density lipoprotein cholesterol (HDL-C) were measured in mg/dl by enzymatic methods (CHOD-PAP, Boehringer Mannheim, Germany). The TC/HDL-C ratio was dichotomized into two groups (<5, ≥5). Body mass index (BMI) was calculated as weight in kilograms divided by height in meters squared, both assessed in a medical examination. For descriptive purposes, BMI was categorized in three groups (<25, 25 - <30, ≥30 kg/m^2^).

Depressed mood was examined by the "Depression and Exhaustion" (DEEX) scale consisting of eight items drawn from the von Zerssen affective symptom check list [25] and using the upper tertile as cut-off point (<11, ≥11). Depressed mood was only available in 10,554 participants.

### Statistical analyses

Mean differences of the UPMS score by categorized variables were assessed by the *t* or *F* test.

Cox regression with different grades of adjustments for cardio-metabolic risk factors was applied to assess the association of the UPMS score with the risk for newly diagnosed type 2 diabetes. The UPMS score was included in the Cox regression models as a continuous variable using a fractional polynomials (FP) transformation approach to find the appropriate score-diabetes risk relation [26]. Adjustments were made for age, sex and survey (model 1) and additionally for the risk factors educational level, and parental history of diabetes (model 2), smoking, alcohol consumption and physical inactivity (model 3), actual hypertension and TC/HDL-C (model 4) and BMI (model 5).

The predictive ability of the Cox models to assess T2DM risk was estimated by the AUC (area under the curve), the IDI (integrated discrimination improvement) and the NRI (net reclassification improvement) following the approach of Pencina et al. [27] as measurements for accuracy of T2DM risk prediction using the SAS macro RECLASSIFICATION_PHREG developed by Mühlenbruch [28].

To assess the robustness of the findings revealed by the Cox regression models, we performed sensitivity and interaction analyses. First, since it cannot be excluded that a UPMS may indicate early signs of a type 2 diabetes disease, we repeated the Cox regression analyses by excluding participants with a follow-up time less than two years (n = 151). Second, to evaluate rather the short term impact of UPSM on T2DM risk, we performed a Cox regression assessing the risk to develop T2DM within 5 years. Third, potential modifications between the UPMS score and age, sex or any of the risk factors stated above were tested by adding the respective interaction term to the fully-adjusted model 5. To account for multiple testing in the interaction analyses, the significance level was corrected by the Bonferroni method leading to a significance level of 0.005 (as ten variables were under concern).

For all statistical analyses, a p value <0.05 was considered to be statistically significant, except for the interaction analyses (p <0.005 following Bonferroni correction). SAS Version 9.2 for Windows (SAS Institute, Cary, NC) was used for all statistical analyses; for applying the FP transformation approach, the SAS macro MFP8 was used [26].

The analysis and the description in this article follow the STROBE guidelines for observational cohort studies [29].

## Results

### Description of study population

A total of 974 newly diagnosed T2DM cases (599 men, 375 women) were observed within a mean follow-up time of 15.6 years indicating 16 newly diagnosed T2DM cases in 1,000 person-years. The (unadjusted) distributions of age, sex and the cardio-metabolic risk factors as well of the UPMS score are shown in Table 1 stratified by T2DM cases and non-cases. Except for smoking and alcohol consumption, the association between classic risk factors with T2DM were highly significant (p value <0.001) with cases being older, more often male and low educated, having higher BMI and TC/HDL-C values, being more frequently physical inactive, having more often a parental history of diabetes and hypertension. Moreover, the mean UPMS score was higher for cases than for non-cases (8.52 versus 7.69).

**Figure 1:**
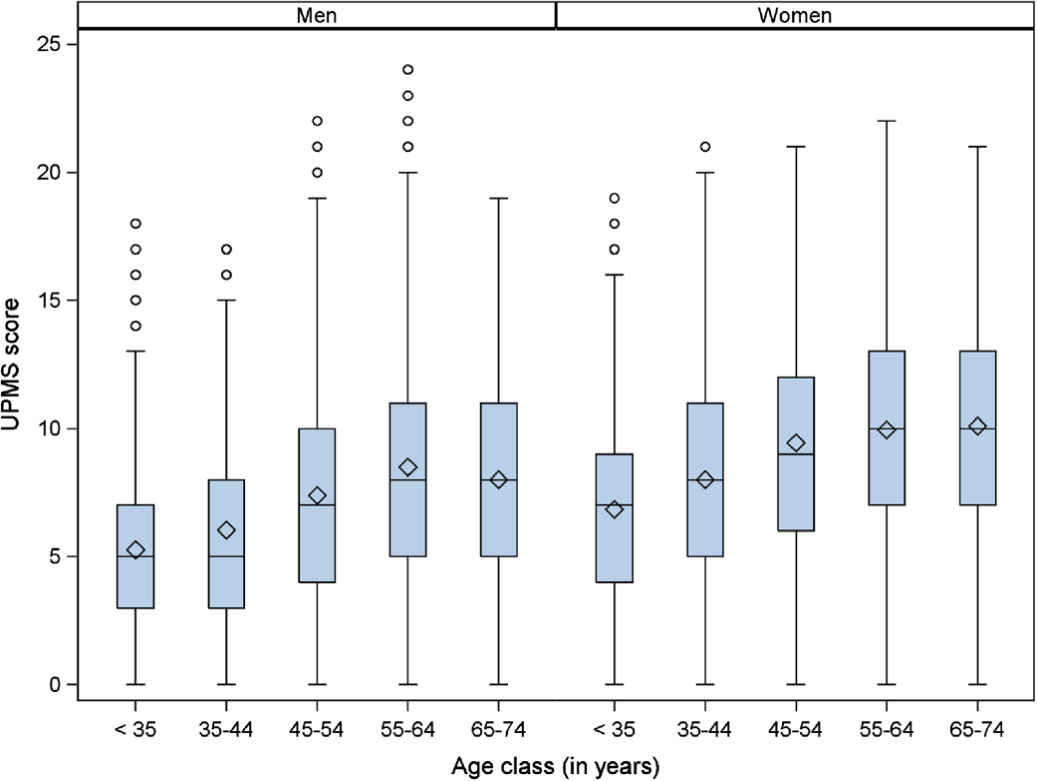
**Distribution of UPMS score by age class in men and women.**

Overall, the UPMS score ranged from 0 to 24 with an average of 7.76 (SD 4.31). A minority of 2.3% (n = 242) of all participants did not report any somatic complaint; for about 10% of the participants, the score was 13 or higher. Figure 1 shows the distribution of the UPMS score by age class separately for men and women. In all age classes, the mean UPMS score was significantly higher in women than in men (p values <0.001). In both sexes, an increasing UPMS score by growing age was observed until age class 55-64 years where a plateau was reached and the UPMS score remained rather stable for participants aged 65-74 compared to 55-64 years.

**Table 1:** **Age, sex and risk factor distribution at baseline in newly diagnosed T2DM cases and non-cases (n = 10,566)**

	T2DM cases	T2DM non-cases	P value
	(n = 974)	(n = 9,592)	
Age (in years)*	52.9 (10.8)	46.1 (13.2)	< 0.001
Male sex	61.5	50.6	< 0.001
Low educational level	80.9	70.1	< 0.001
Parental history of diabetes			< 0.001
No	48.1	64.4	
Yes	29.5	18.1	
Unknown	22.5	17.6	
Current smoker	29.8	27.3	0.10
Alcohol consumption			0.52
No	28.5	28.0	
Moderate	43.4	45.3	
High	28.0	26.7	
Physical inactivity	66.0	55.8	< 0.001
Actual hypertension	59.8	33.5	< 0.001
TC/HDL-C*	5.49 (2.16)	4.39 (1.77)	< 0.001
BMI (in kg/m^2^)*	29.7 (4.5)	26.1 (3.9)	< 0.001
UPMS score*	8.52 (4.62)	7.69 (4.27)	< 0.001

*mean (standard deviation).

BMI: Body mass index; TC/HDL-C: Total cholesterol/High density lipoprotein cholesterol; UPMS: Unspecific premonitory symptom.

Table 2 gives the mean UPMS score (with standard deviation) for each cardio-metabolic risk factor. Compared to their respective counterparts, the mean score was significantly higher in women, in older age groups, in less educated and obese subjects, in non-smokers and non-alcohol consumers and in physically inactive subjects, in subjects with known or unknown parental history of diabetes and hypertension (p values <0.001).

**Figure 2:**
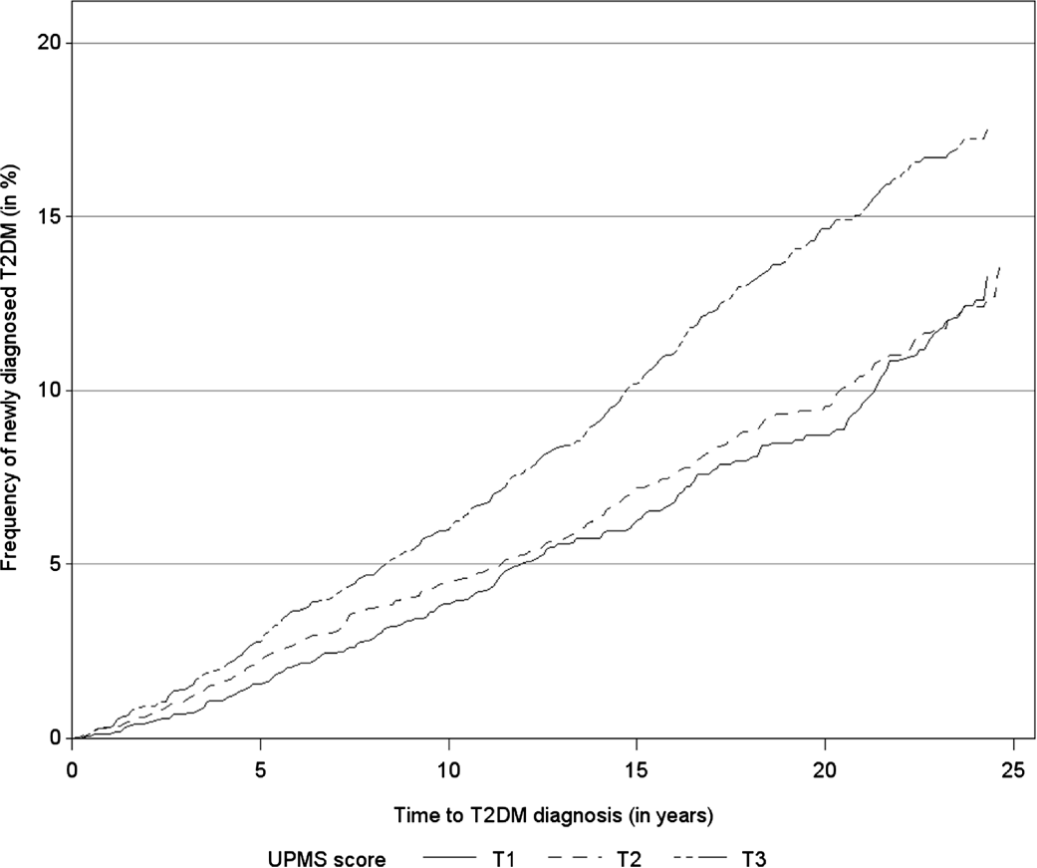
**Frequency of newly diagnosed of T2DM by UPMS score tertiles (T1, T2, T3) estimated by Kaplan-Meier curves**.

The frequency of newly diagnosed T2DM in the UPMS score tertiles were estimated by Kaplan-Meier curves (Figure 2) indicating higher risks for T2DM in the upper tertile (T3) compared to the lower and middle tertiles (T1 and T2).

**Table 2:** **Risk factors and mean UPMS score (n =10,566)**

Risk factor	Category	UPMS score	P value
		Mean (standard deviation)	
All		7.76 (4.31)	-
Educational level	Low	8.24 (4.40)	< 0.001
	High	6.59 (3.83)	
Parental history of diabetes	No	7.48 (4.20)	< 0.001
	Yes	8.13 (4.35)	
	Unknown	8.38 (4.54)	
Current smoker	No	7.91 (4.28)	< 0.001
	Yes	7.37 (4.36)	
Alcohol consumption	No	8.46 (4.58)	< 0.001
	Moderate	7.52 (4.15)	
	High	7.44 (4.20)	
Physical inactivity	No	6.99 (4.05)	< 0.001
	Yes	8.35 (4.40)	
Actual hypertension	No	7.59 (4.19)	< 0.001
	Yes	8.07 (4.49)	
TC/HDL-C	< 5	7.81 (4.24)	0.14
	≥5	7.67 (4.47)	
BMI (kg/m^2^)	< 25	7.43 (4.13)	< 0.001
	25- <30	7.80 (4.34)	
	≥30	8.45 (4.55)	

BMI: Body mass index; TC/HDL-C: Total cholesterol/High density lipoprotein cholesterol; UPMS: Unspecific premonitory symptom.

### Association of the UPMS score with the risk for newly diagnosed T2DM

Cox regression analyses using FP transformation revealed a linear relation between the UPMS score and newly diagnosed T2DM risk in all models. Adjusted for age, sex and survey, the risk for newly diagnosed T2DM increased with a hazard ratio (HR) of 1.03 (95% CI 1.01-1.04, p <0.001) per each UPMS score unit increase as shown in Table 3. Additional adjustments for cardio-metabolic risk factors attenuated this effect but significance remained. The fully-adjusted model 5 estimated a HR of 1.02 (95% 1.01-1.04), p value = 0.01); calculations based on this estimation revealed for example that the T2DM risk of an individual with a score at the 75^th^ percentile (=11) compared to an individual with a score value at the 25^th^ percentile (=5) increased by 12%.

**Table 3:** **Association of the UPMS score with risk for newly diagnosed type 2 diabetes estimated by Cox regression: Hazard ratio (95% confidence interval) and p value (n =10,566)**

	HR	P value
	(95% CI)	
Model 1: Age, sex and survey	1.03	< 0.001
	(1.01-1.04)	
Model 2: M1 + educational level and parental history of diabetes	1.02	0.001
	(1.01-1.04)	
Model 3: M2 + smoking, alcohol consumption, physical inactivity	1.02	0.02
	(1.00-1.03)	
Model 4: M3 + hypertension, TC/HDL-C	1.02	0.005
	(1.01-1.04)	
Model 5: M4 + BMI	1.02	0.01
	(1.01-1.04)	

HR: Hazard ratio; CI: Confidence interval; UPMS: Unspecific premonitory symptom.

An analysis of the predictive ability of UPMS for T2DM risk indicated very low improvement by adding the UPMS score. Regarding T2DM risk within 10 years, the AUC was 0.7995 in the model without and 0.7999 with the UPSM score in the model adjusted for all cardiovascular risk factors (model 5). Similar findings were observed by estimating IDI and NRI.

### Sensitivity and interaction analyses

Excluding participants with a follow-up time of less than two years (71 cases, 80 non-cases) revealed comparable hazard ratios (model 1: HR: 1.03, 95% CI: 1.01-1.04, model 5: HR: 1.02, 95% CI: 1.00-1.03) and p values (model 1: <0.001 and model 5: 0.02) for the UPMS score. Assessing the risk to develop T2DM within five years led to similar and significant findings with a HR of 1.04 (95% CI 1.01-1.07, p value 0.006) in model 1 and a HR of 1.03 (95% CI 1.00-1.06, p value 0.03) in model 5.

Including additionally depressed mood in the fully-adjusted model decreased the hazard ratio of the UPMS score to 1.01 (0.99-1.03, p value 0.18). Stratified analyses estimated for the first model for the score a hazard ratio of 1.02 (95% CI 1.00-1.04, p value 0.10) in the non-depressed mood group (n = 6,769) and 1.06 (95% CI 1.03-1.09, p value <0.001) in the group with depressed mood (n = 3,785).

Regarding potential interactions, we could not detect any significant modification of this effect by age, sex or any of the cardio-metabolic risk factors after correction for multiple testing. However, suggestive interactions were found for TC/HDL-C and BMI with a stronger effect of the UPMS score on T2DM risk in participants with a higher compared to a lower TC/HDL-C (p value 0.03) and in lower BMI compared to higher BMI values (p value 0.04) in fully-adjusted models.

## Discussion

### Overall findings

In this long-term prospective population based study, we found that apparently healthy participants reporting a distinct pattern of unspecific somatic symptoms at baseline experienced a substantial increase in newly diagnosed T2DM risk over the observation period, even after controlling for classical cardio-metabolic risk factors. An unspecific premonitory symptom (UPMS) pattern has been identified as a premonitory prodromal sign of a serious chronic disease condition long before the onset of the disease particularly in acute coronary syndromes [1, 2] – however, to the best of our knowledge – this has not been shown before in the case of the onset of a T2DM.

The somatic symptoms which have been captured by the 8-item Somatic Symptom Scale-8 (SSS-8) in the present investigation have only a negligible overlap with the symptoms acknowledged as being "classic" early diabetes signs by the ADA and IDF [13, 14]. The clinical presentation of early diabetes symptoms is usually related to polyuria, polydipsia, weight loss, blurred vision and sometimes polyphagia whereas the somatic symptom pattern assessed in the present investigation compiles the following symptoms: stomach or bowel pain, back pain, pain in the joints, headaches or pressure in the head, temporary shortness of breath, dizziness, feeling tired and insomnia. The present investigation gives indications to consider this particular array of somatic symptoms as premonitory signs of T2DM within a long time window before clinical recognition.

### Pathophysiological explanations

Although an exact evaluation of the pathophysiological pathways is beyond the scope of the present study, we assume three major possible explanations for underlying mechanisms that may lead to an association between the UPSM score and newly diagnosed T2DM risk in our study population.

First, one may suspect that an elevated burden of unspecific premonitory symptoms is a prodromal sign of an already existing but still undiagnosed T2D. This is not unlikely as it is well established that the risk of T2DM patients developing microvascular complications often predates the point of clinical recognition by several years [9, 10]. However, in a sensitivity analysis excluding subjects with a follow-up time of less than two years, the impact of the UPMS score on newly diagnosed T2DM risk remained stable with very similar hazard ratios which renders this explanation as unlikely.

Second, we observed in the present investigation that subjects with high UPMS scores were more likely to experience hypertension, obesity and physical inactivity. It is well established that subjects at risk to develop T2DM are likely to cluster cardiovascular risk factors [9] with subsequent higher risks of the onset of CVD and diabetes-related complications. Nevertheless, the impact of the UPMS score on newly diagnosed T2DM risk remained significant independent from the cardio-metabolic risk profile indicating that an elevated UPMS had its own contribution to T2DM risk assessment. The association of UPMS and CVD risk factors not only reflects an unfavourable state of health contributing to the onset of diabetes in the future but also points to a sustained subclinical heightened inflammatory activity as a common link of these risk conditions. Indeed, inflammatory activity has been shown for untreated hypertension [30–32], obesity [31, 33] and physical inactivity [34, 35]. Inflammation, in turn, can trigger behavioural consequences which resemble symptoms which are at least in part captured by the SSS-8 (e.g. dizziness, feeling tired and insomnia) [36, 37].

Third, the increased preoccupation and awareness of bodily symptoms patterns is likely to cause chronic stress particularly as the lack of a "medical explanation" for unspecific symptoms often leads to feelings of uncertainty, to rejection from the side of the physician with subsequent symptom amplification and persistence of symptoms – yet without any prospects of successful treatment. Furthermore, there is a large body of evidence suggesting that the sustained experience of a high somatic symptom burden is also strongly associated with adverse mental health related conditions such as depression, anxiety, sleeping disturbance or low self-perceived health [38–40]. Permanent psychological distress and consequently in chronic stress conditions has been shown to contribute to adverse metabolic dysregulations [41, 42] and furthermore to increased T2DM risk [22, 43–46]. Thus, our findings may be explained partly by the relation between elevated unspecific symptom burden and adverse chronic stress conditions. Particularly, depressed mood has been proven as a risk marker for the onset of T2DM. Mezuk et al. (2008) performed a meta-analysis including 13 studies with 6,916 incident T2DM cases and revealed that depression increased the risk for T2DM by 60% [47]. The association between depression and insulin resistance was shown to be small, however robust [48]. In the present investigation, analyses stratified for depressed mood revealed rather similar risk estimates for the UPSM score in participants without and with depressed mood (HR 1.02 and 1.06) in a model adjusted for age, sex and survey. However, when depressed mood was added to the fully-adjusted model in the total sample, the effect of the UPMS score decreased to 1.01 and significance vanished (p value 0.18). Also depressed mood did not reach significance in this model (p value 0.15). As both variables were highly correlated, inclusion of the score and depressed may over-adjust the Cox model. Therefore, as a practical consequence, when dealing with patients potentially at risk, physicians may assess the symptom count of premonitory signs or may screen for depressed mood – the findings will be most likely very similar.

### Strengths and Limitations

The strengths of the present study are the prospective design, the large sample size based on a random sample drawn from the general population and the availability of a large set of risk factors which were scrutinized by standardized and quality-controlled assessments. Additionally, as far as we know, this is the first prospective study investigating the association of an unspecific premonitory symptom pattern with newly diagnosed type 2 diabetes risk in a population-based sample. The distribution of risk factors at baseline in newly diagnosed T2DM cases and non-cases as shown in Table 1 as well as the impact of risk factors on T2DM risk as estimated by the Cox regression analyses (data not shown) were as expected and comparable to previous studies.

One major limitation is that we cannot assess whether the symptom pattern was caused by specific chronic disease conditions. If a strong relation between unspecific symptom patterns and chronic diseases is assumed, a constant increase of symptom reporting by growing age would be expected (as the prevalence of chronic diseases increase by age). However, symptom reporting remained stable after in participants aged 55 years or older as shown in Figure 1 for our study population indicating that a causation of symptom patterns mainly by chronic diseases may not be assumed. Moreover, our findings may be affected by other incident diseases within the follow-up period which could not be assessed in the present study population. The improvement in predictive ability for T2DM risk was rather low when the UPMS score was added to the cardiovascular risk factors in the Cox regression (model 5). Nevertheless we think that our findings give important indications to consider a particular array of somatic symptoms as premonitory signs of T2DM within a long time window before clinical recognition. The AUC in the present study were around 0.80 which was in the range of AUCs (0.76 to 0.81) estimated in a recently published validation study of 12 existing T2DM prediction models [49]. Furthermore, it cannot be excluded that participants reporting no history of diabetes at baseline examination suffer from an undiagnosed diabetes or a prediabetic state. Finally, UPMS is currently not an inherent part of data collection in routine setting. However, the documentation of a UPSM pattern consisting of several unspecific somatic symptoms would be easy to assess in routine settings with limited time costs.

## Conclusions

This prospective population-based study found a substantial association of an elevated burden of unspecific premonitory symptoms with T2DM long before the onset and independent of established cardio-metabolic risk factors. Further research is needed to confirm or refute our findings and to obtain insight in potential underlying pathophysiological mechanisms.
